# Validity and Reliability of the Turkish Version of the Celiac Dietary Adherence Test

**DOI:** 10.3390/medicina61122239

**Published:** 2025-12-18

**Authors:** Yeliz Serin, Gözde Ede İleri, Pelin Akın, Jurgita Andruškienė, Simona Grigaliūnienė, Darius Drungilas, Žydrūnas Lukošius, Mindaugas Kurmis

**Affiliations:** 1 Department of Nutrition and Dietetics, Faculty of Health Sciences, Çukurova University, Adana 01330, Türkiye; 2 Department of Nutrition and Dietetics, Faculty of Health Sciences, Çankırı Karatekin University, Çankırı 18100, Türkiye; gozdeede@karatekin.edu.tr; 3 Department of Statistics, Faculty of Science, Çankırı Karatekin University, Çankırı 18100, Türkiye; pelinakin@karatekin.edu.tr; 4 Klaipeda University, 92294 Klaipėda, Lithuania; jurgita.andruskiene@ku.lt (J.A.); simona.grigaliuniene@ku.lt (S.G.); darius.drungilas@ku.lt (D.D.); zydrunas.lukosius@ku.lt (Ž.L.); mindaugas.kurmis@ku.lt (M.K.)

**Keywords:** celiac disease, gluten-free diet, celiac dietary adherence test, validation, adult

## Abstract

*Background and Objectives*: In clinical practice and research, it is necessary to use a standardized measurement tool to accurately determine dietary adherence in adults with celiac disease. The aim of this study is to validate the Celiac Dietary Adherence Test (CDAT) in the Turkish patients with celiac disease. *Materials and Methods*: The CDAT was translated using forward-backward translation method, reviewed by experts, and tested for comprehensibility. The final revised version of the CDAT was completed by 97 adults diagnosed with celiac disease and following a gluten-free diet. Construct validity was assessed using exploratory factor analysis (EFA) and confirmatory factor analysis (CFA). Reliability was evaluated via internal consistency (Cronbach’s alpha) and test–retest reliability (Pearson correlation) after re-administration within 2 weeks. *Results*: Exploratory factor analysis yielded a Kaiser-Meyer-Olkin (KMO) value of 0.69, with Bartlett’s test of sphericity being significant (*p* < 0.001). EFA indicated a single-factor structure, explaining 55% of the total variance. CFA showed acceptable fit indices for the model (χ^2^/df = 1.45, CFI = 0.93, TLI = 0.90, RMSEA = 0.07, GFI = 0.94, AGFI = 0.88). Reliability analyses indicated Cronbach’s alpha of 0.70, and a test–retest correlation of 0.92. Items 5 and 6 were removed from the Turkish version of the CDAT because their factor loadings were below 0.40. *Conclusions*: The Turkish CDAT is valid and reliable for rapid, standardized assessment of adherence to a gluten-free diet in adults with celiac disease.

## 1. Introduction

Celiac disease (CD) is a chronic autoimmune enteropathy characterized by total or partial atrophy of the small intestinal villi and inflammation of the mucosa, triggered by dietary gluten intake in genetically predisposed individuals [1,2]. The global prevalence of biopsy-diagnosed CD is approximately 0.7% [3]. In Turkey, the prevalence varies between 1% and 0.3 per thousand, and according to data from the Ministry of Health’s Health Information Systems, the total number of diagnosed celiac patients was 166.614 as of November 2023 [4]. The only treatment for CD is a gluten-free diet, which is requires the elimination of gluten-source foods such as wheat, rye, barley and, in some cases, oats [5]. Studies indicate that adherence to a gluten-free diet varies between 42% and 91% [1,6,7]. Adherence to a gluten-free diet plays a crucial role in alleviating clinical symptoms, preventing CD-related complications and improving nutritional status and quality of life [1,8]. The literature shows that inadequate adherence to a gluten-free diet is associated with decreased bone mineral density and osteoporosis, an increased risk of malignancy, especially lymphoma, and persistent micronutrient deficiencies such as iron, folate, and vitamin B_12_ [9,10].

During the gluten-free diet adaptation process, individuals must make lifelong changes to their daily dietary habits. Therefore, adherence to the gluten-free diet can be laborious, expensive, and socially restrictive [11]. Studies of individuals with CD have shown that gluten-free foods and packaged products are often more costly than their gluten-containing counterparts. Additionally, taste and texture may be of lower quality, with limited product availability and variety, and sometimes inadequate labeling information. There is also concern about the production conditions of unpackaged and open-air foods [12,13]. In a study by Dimidi et al., it was determined that individuals with inadequate adherence to the gluten-free diet had difficulty preparing gluten-free foods, were uncertain about safe environments for food preparation outside the home, and found it difficult to eat at school, university, workplaces and with friends or family, which generally hindered adherence to the diet [1]. The cost of following a gluten-free diet and its social restrictions can lead to negative outcomes, such as anxiety and depression, and may cause patients to fear gluten contamination [14]. Studies have also emphasized that difficulty adhering to a gluten-free diet increases the likelihood of anxiety, depression, and eating disorders among individuals with CD [2,11,15,16]. Moreover, following a gluten-free diet may contribute to disordered eating attitudes and behaviors in some people [17]. Skipping meals, restricting certain foods, and concerns about food safety and cross-contamination are among eating disorder symptoms [18,19]. Although gluten-free diet is physiologically beneficial for people with CD, its restrictive nature can affect quality of life, promote neophobia, and lead to maladaptive eating behaviors, including disordered eating patterns [5,17,18,20,21].

In clinical practice, adherence to a gluten-free diet is assessed using serological tests (tissue anti-transglutaminase), histopathological findings (small intestinal biopsy) and/or detailed nutritional history [22]. However, serological and histopathological evaluations are costly and invasive and require experienced personnel. Consequently, validated scales to assess adherence to a gluten-free diet in adults with CD are considered more feasible than invasive and costly methods used by health professionals [23,24]. The literature includes several self-report instruments other than the CDAT (Celiac Dietary Adherence Test) for assessing dietary adherence in celiac patients, such as the Gluten-Free Score and TPB-based questionnaires [25,26]. However, CDAT was preferred in our study due to its brevity, suitability for clinical/practical use, and psychometric validity. This choice is supported by studies demonstrating the widespread use of CDAT in both research and clinical settings and its adaptability to different cultures [27]. The CDAT is a concise self-report instrument developed for this purpose [28]. The aim of this study was to validate the CDAT to Turkish because there is no gluten-free diet adherence scale for adults with CD in Türkiye.

## 2. Materials and Methods

### 2.1. Study Design and Participants

This methodological study was conducted with adult CD patients in Türkiye. Inclusion criteria were age ≥ 18 years, university undergraduate status, biopsy-proven CD, and adherence to a gluten free diet. Exclusion criteria included having any psychological disorder and/or chronic disease requiring medical nutritional therapy, no active gastrointestinal complaints in the last three months, and any eating disorder. Information was collected using self-reported screening questions tailored to DSM-5 diagnostic criteria, a widely accepted method in the literature. Clear instructions were provided to participants to prevent misrepresentation and were designed to reflect the diagnostic features of these disorders [29].

### 2.2. Ethical Considerations

The study was approved by the Çankırı Karatekin University Health Sciences Scientific Research and Publication Ethics Board (protocol code: 08, date: 29 January 2025) and conducted in accordance with the principles of the Declaration of Helsinki. Written informed consent was obtained from all participants prior to inclusion.

### 2.3. Data Collection

Data were collected between 15 April 2025 and 15 June 2025 using an online questionnaire prepared with Google Forms. The online survey was distributed to adult patients with CD via links to celiac-related societies on social media applications (Instagram and WhatsApp) frequently used in daily life. We reached approximately 1000 adult celiac patients on these platforms. The response rate was approximately 10%. Information regarding the purpose and content of the study was detailed on the first page of the online consent form outlining the study aim, voluntary participation, confidentiality, and contact details. Volunteering adult celiac patients checked ‘I agree’ to the question “Do you voluntarily participate in this study?” on the first page of the online survey and then proceeded to the questions.

The online questionnaire comprised four sections: sociodemographic factors, disease-related information, anthropometric measurements, and CDAT. The first section captured age of the patients, age at diagnosis, gender, and marital status. The second section included information related to dietitian visits, self-reported adherence to the gluten-free diet (GFD), adherence level outside the home, and expenditure on GFD. The third section collected body weight and height measurements. The final section contained the CDAT items.

Body weight and height were obtained from measurements recorded by healthcare professionals during routine medical examinations at the hospital. BMI was calculated as weight (kg) divided by height (m^2^) and categorized according to the World Health Organization (WHO) classifications: normal weight (18.5 ≤ BMI < 25.0 kg/ m^2^), overweight (25.0–29.9 kg/ m^2^) and obesity class I (30.0–35.0 kg/m^2^).

#### Celiac Dietary Adherence Test

Celiac Dietary Adherence Test (CDAT) was developed to assess adherence to a gluten-free diet and contains seven items [28]. Each item is evaluated on a five-point Likert scale. The total CDAT score is calculated by averaging the seven items and ranges from 7 to 35. A total score < 13 indicates good adherence, scores between 13 and 17 indicate moderate adherence, and scores > 17 indicate poor adherence.

### 2.4. The Translation and Adaptation

Permission to translate and adapt the original scale for validity and reliability analysis was obtained from Lefler [28], the corresponding author and copyright holder of the original instrument, via email.

The translation process of the original CDAT followed three main steps: forward translation, back translation, and evaluation of item understandability by five academics who are experts in nutrition and dietetics. First, the scale was translated from English to Turkish by researchers in the Department of Nutrition and Dietetics during the forward translation phase. The two translations were compared to prepare a common version of the scale. The items were then translated back into English by a researcher in statistics who was not familiar with the items in the scale during the back-translation phase. This back-translated version was sent to native English-speaking researchers involved in the study for evaluation. After their evaluation, the subsequent version, along with two forward-translated versions and the original version, was sent to five researchers in the fields of CD and nutrition. Each expert completed a form using a four-point Likert scale (1 = not at all appropriate to 4 = completely appropriate) to assess the appropriateness, clarity, and comprehensiveness of each item. Item-level content validity index (I-CVI) was calculated based on the proportion of experts rating an item as “appropriate” or “very appropriate.” The I-CVI obtained was 0.81. Based on these evaluations, the researchers refined the item translations to develop the most comprehensible and culturally acceptable version. A pilot study with 15 adults with CD was conducted to evaluate the understandability and social relevance of the CDAT items using the final version of the scale.

### 2.5. Construct Validity

To assess construct validity, the Kaiser-Meyer-Olkin (KMO) measure and Bartlett’s test of sphericity were used for exploratory factor analysis (EFA). Factor loadings were examined, and the model’s fit was further evaluated with confirmatory factor analysis (CFA) using indices including Comparative Fit Index (CFI), Tucker–Lewis Index (TLI), Adjusted Goodness-of-Fit Index (AGFI), Root Mean Square Error of Estimation (RMSEA, the Goodness-of-Fit Index (GFI), Normed Fit Index (NFI), and the chi-square to degrees of freedom ratio (x^2^/sd).

### 2.6. Reliability

Internal consistency of CDAT was assessed with Cronbach’s alpha. Inter-rater reliability was evaluated by comparing questionnaire results from two independent raters who assessed the same participants (n = 97) on the same day. Intra-rater reliability (test–retest) was measured by re-administering the questionnaire to the same participants after an interval of approximately two weeks [30]. Both inter-rater and intra-rater reliabilities were analyzed using intraclass correlation coefficients (ICCs), with values ≥ 0.75 indicating excellent reliability. Test–retest reliability was also assessed using the Pearson correlation coefficient. The ICC was used to capture temporal stability more comprehensively by accounting for inter- and intra-individual variance [31]. Using both indices together provides a stronger methodological framework for reliability assessment because they are complementary.

### 2.7. Statistical Analysis

To determine the sample size for validity and reliability studies, it is recommended to include individuals five or ten times the number of items on the scale [32,33]. The CDAT contains seven items, so at least 35 or 70 individuals meeting the inclusion criteria should participate to represent the population. Accounting for potential attrition, the literature recommends increasing the sample size by 5% [34]. Accordingly, by including 97 patients in this study, a 35% larger sample size was achieved.

Descriptive statistics were summarized as mean ± standard deviation (x ± SD) and counts and percentages. The Pearson correlation test was used to assess test–retest reliability. Significance was assessed at *p* < 0.05.

Validity and reliability analyses were conducted on data obtained from the scale. For structural validity, Exploratory Factor Analysis (EFA) was used to reveal the factor structures, while Confirmatory Factor Analysis (CFA) and item discrimination analyses were used to verify the identified factors. The Kaiser-Mayer-Olkin (KMO) measure and Bartlett’s test of sphericity were applied to determine the suitability of the data for EFA. For CFA, fit indices included CFI, TLI, AGFI, RMSEA, GFI, NFI, and x^2^/sd.

The item removal process was conducted sequentially (iteratively). At each step, the item with the lowest item–total correlation whose removal increased Cronbach’s alpha was removed from the scale, followed by the re-running factor analyses and reliability analyses.

Reliability analyses used Cronbach’s Alpha for internal consistency, item total correlations, and the test–retest method. Item–total correlations evaluate each item’s contribution to the scale while factor loadings indicate the degree to which each item represents the corresponding factor. Internal consistency was considered acceptable when Cronbach’s alpha ≥ 0.70 [35]. These analyses were interpreted together to assess the construct validity and reliability of the scale. For test–retest results, the Intraclass Correlation Coefficient (ICC) was calculated, the value of 0.75 and above was considered acceptable. Statistical Package for the Social Sciences (SPSS 25.0) and R Programming (4.1.3) were used for statistical analysis.

## 3. Results

### 3.1. Patients Characteristics

The study included 97 patients: 78 females and 19 males. The mean age was 35.4 ± 11.64 years. The mean age at diagnosis was 28.0 ± 12.62 years. Most of patients had normal BMI. The sociodemographic characteristics of the adult patients with CD are summarized in Table 1.

Dietitian visits, adherence to the gluten-free diet (GFD), and income spent on gluten-free foods are presented in Table 2. The majority (62.9%) had never consulted a dietitian for guidance on implementing their GFD. However, over half (51.5%) reported always adhering to a GFD when outside the home. Regarding income spent on following a GFD, participants reported that they could allocate only 25% of their income to gluten-free products.

### 3.2. Item Analysis

The mean and standard deviation for each item of the CDAT are summarized in Table 3.

### 3.3. Results of Construct Validity Analysis

The Kaiser-Meyer-Olkin (KMO) measure was 0.69, and Bartlett’s test indicated that the correlation matrix was not a unit matrix (χ^2^(21) = 107.50, *p* < 0.001). These results suggest that the sample is adequate for factor analysis. In the initial analysis, a single-factor model was examined for the seven-item scale; this factor explained 35.0% of the total variance. Factor analysis was applied to assess the scale’s construct validity. Factor loadings ranged from 0.394 to 0.846, item 5 with a factor loading below 0.40 was removed from the scale (Table 4).

The scale’s goodness-of-fit indices and acceptability values are presented in Table 5. Specifically, χ^2^/df = 1.452 falls within acceptable limits (<5). GFI = 0.941, AGFI = 0.881, CFI = 0.931, and TLI = 0.896 are within acceptable ranges. The RMSEA of 0.068 indicates a moderately acceptable fit, while the NFI = 0.819 is slightly below the acceptable threshold. Overall, model fit was acceptable, although the NFI remained below the commonly recommended value. Model fit was found to be root mean square residual (RMSR) = 0.12, and the chi-square test was significant (χ^2^(14) = 63.52, *p* < 0.001).

As a result of item analysis, items 5 and 6 were found to reduce the scale’s reliability and to have low item-total correlations (Table 6). After removing these two items, factor analysis was repeated on the five-item scale. In the new model, the factor explained 55.0% of the total variance, and factor loadings ranged from 0.56 to 0.79. Model fit was indicated by RMSR = 0.15, the chi-square test was significant (χ^2^(10) = 43.79, *p* < 0.001), and the fit index was 0.78. Internal consistency increased to Cronbach’s alpha = 0.70. These findings indicate that the five-item version of the scale is psychometrically acceptable and provides adequate validity and reliability under a single-factor structure.

During the item reduction process, items 5 and 6 were removed due to low factor loadings and item-total correlations. However, these items reflect the psychological and risk perception dimensions of the scale. Their removal narrows the conceptual scope of the shortened version and causes it to differ from the original CDAT in certain respects. The new five-item version emphasizes more behavioral and cognitive aspects, while the psychological and risk perception dimensions are relatively weak. This should be considered in interpreting the scale, and reassessment of these dimensions is recommended in future studies.

### 3.4. Results of Reliability Analysis 

The internal consistency of the Turkish CDAT was confirmed by Cronbach’s alpha, yielding a value of 0.70, thereby supporting its acceptable reliability (Table 7). Item–total correlations ranged from 0.187 to 0.616, all items except two surpassed the acceptable threshold (≥0.30).

The test–retest reliability was assessed after re-application of the scale, using the intraclass correlation coefficient (ICC), and a dependent samples *t*-test to evaluate the scale’s unidimensionalilty (Table 8). No significant difference was found between test and retest values (*p* > 0.05). The CDAT demonstrated high reliability with ICC = 0.950 (95% CI: 0.926–0.966), and the ICC was significant at the *p* < 0.001. The test–retest correlation was r = 0.95 (*p* < 0.001), indicating high stability over a 2-week interval. The Cronbach’s alpha for the total score of the Turkish-adapted CDAT was 0.974. These findings demonstrate excellent reliability of the Turkish CDAT questionnaire.

## 4. Discussion

Adherence to the gluten-free diet (GFD) is crucial for preventing symptoms, enhancing quality of life, and reducing healthcare costs for adult patients with CD [36]. Using an incorrect method to confirm GFD adherence may pose a risk CD patients’ diets. Therefore, we believe that this scale, which we are validating, will be beneficial for assessing adherence to gluten-free diet treatment in adult patients with CD more quickly and at lower cost [24].

Translational validity and reliability of GFD adherence tests are crucial for accurate evaluation in the treatment of celiac disease (CD). These characteristics ensure that assessments such as the Celiac Dietary Adherence Test (CDAT) translate research findings into clinical practice and correlate favorably with gold standards such as biopsies, as well as with commonly used tests like serology and gluten immunogenic peptides (GIPs) [24,25]. In addition, reliability and validity of CDAT across different languages, support global use [37,38]. On the other hand, gluten free diet adherence tools can reveal barriers and gaps contributing to underdiagnosis, including poor disease awareness among physicians and/or patients, limited access to diagnostic resources, inappropriate use or interpretation of the serological tests, lack of standardized diagnostic and endoscopic protocols, and insufficient expertise in histopathological interpretation and the factors that influence GFD adaptation [39]. For this reason, subjective assessments (questionnaires) and objective assessments (GIP) should be considered to evaluate GFD exposure, particularly when an expert dietitian is not available [40].

In subgroups with comorbidities, specific difficulties with GFD adherence necessitate focused assessment. For example, children with type 1 diabetes and CD require more nutritional counseling and follow-up to emphasize GFD adherence [41]. Similarly, patients on a GFD who experience functional gastrointestinal symptoms benefit from diet adherence validation tests [42]. Thus, these tests may aid in the adoption and maintenance of GFD across diverse CD populations.

Gluten-free diet assessment tools or scales are widely used by health professionals [24,28,43]. However, there is no specific validated scale to evaluate dietary adherence of adults with CD in Türkiye. Therefore, the aim of this study was to validate the Celiac Dietary Adherence Test (CDAT) developed by Leffler et al. into Turkish [28]. The CDAT, validated for Turkish, demonstrated good internal consistency. Factor analysis revealed a single factor. Internal consistency was acceptable, indicated by a Cronbach’s alpha of 0.70 suggesting that our scale is reliable. Factor analyses confirmed the underlying structure of the scale, and test–retest results showed good stability.

In this study, although the sample size used for confirmatory factor analysis was considered adequate according to the “5–10 times the number of items” rule commonly adopted in measurement tool development and adaptation studies, it is relatively limited in terms of CFA statistical power, especially after the item reduction. The NFI value fell slightly below the recommended threshold, which is consistent with literature indicating that this index can yield more variable results in small samples [44]. However, indices such as GFI, CFI, TLI, and χ^2^/df, which are less affected by sample size, were at acceptable levels, and the RMSEA was within a reasonable range, indicating a satisfactory overall fit.

The Cronbach’s alpha coefficient of 0.70–0.80 suggests the scale is reasonably reliable, with values above 0.80 indicating high reliability [45]. The Cronbach’s alpha coefficient reflects internal consistency of the items within the scale [46]. In our study, while Cronbach’s alpha based on item responses was modest (0.65–0.69), the high alpha (0.974) obtained from test–retest analysis reflects temporal stability rather than item-level internal consistency. The very high (α = 0.974) retest Cronbach’s alpha can be interpreted as an indicator of stability over time, as responses to items in the second application of the scale tend to be more consistent. In test–retest designs, increased familiarity with the items, learning effects, and repetition of cognitive processes can raise consistency [47]. Moreover, Cronbach’s alpha measures only internal consistency and high alpha values reflect this aspect of reliability [35,48]. Thus, the high retest alpha supports the scale’s stability over time, though the item-level reliability may require improvement. The DFA results show that the NFI value (0.819) remains below the acceptable threshold, which may relate to sample size limitations and the single-factor structure of the scale.

Reliability results are comparable to other studies. Similar findings have been observed in other language adaptations such as Spanish version (Cronbach’s alpha = 0.809) [37], the Persian version (0.71) [49], and the Swedish version (0.716) [50]. The reliability results of the present study are comparable to those of the original CDAT, which reported a Cronbach’s alpha of 0.716 [28,50]. Similar findings have also been reported in other language adaptations supporting the scale’s cross-cultural applicability [50,51]. Minor differences in factor loadings or internal consistency values may be attributed to cultural and linguistic variations and to sample characteristics such as disease duration and education level [52].

The internal consistency coefficient (Cronbach’s alpha) obtained in this study was lower than some values reported in the literature [50]. This difference may reflect factors such as cultural context, sample characteristics, and item reduction. Furthermore, the measurement invariance of the shortened version with the original scale was not tested in this study. Therefore, examining construct equivalence and measurement invariance across language versions in future research will strengthen the scale’s usability for international comparisons.

The validation of the Turkish CDAT provides an important contribution to CD management in Türkiye. The availability of a standardized and culturally adapted tool enables clinicians and dietitians to assess adherence to the GFD more accurately and systematically. This may help identify patients at risk of poor adherence and inform targeted nutritional counseling. Moreover, the instrument can be used in future research to explore the relationship between dietary adherence and clinical outcomes, such as quality of life, nutritional status, and disease complications [27,43].

This study has several strengths. It followed rigorous translation and cultural adaptation procedures, included a sufficient sample size for factor analyses, and assessed multiple aspects of validity and reliability. However, certain limitations should be acknowledged. Participants were recruited from a limited number of centers, which may restrict generalizability. Test–retest reliability was assessed in a smaller subsample, and the scale was validated only among adults; applicability in pediatric populations remains to be investigated. Because the study was conducted online, laboratory and clinical findings of the patients could not be accessed. Future research should evaluate the responsiveness of the Turkish CDAT in intervention studies.

## 5. Conclusions

Translational validity and reliability of gluten-free diet (GFD) adherence tests are crucial in the treatment of celiac disease (CD), for global applications, and for evaluating the impact of comorbidities and quality of life. The Turkish version of the CDAT demonstrated acceptable internal consistency and evidence of construct validity. It is a practical and easy-to-administer instrument for assessing self-reported adherence to a GFD among Turkish adults with CD and can serve as a useful tool in both clinical practice and research. Validated in Turkish, the CDAT facilitates communication and assessment of patients’ adherence to a gluten-free diet by healthcare professionals and researchers. Despite cultural and linguistic variations, the CDAT shows reliability comparable to other versions of scales. Studies using this scale are expected to contribute to the scientific literature by reflecting adherence to a gluten-free diet.

However, the study’s main limitations include a relatively small sample size and limited number of centers, which may restrict generalizability. Although the CDAT demonstrated reliability and construct validity in this study, criterion validity was not assessed. Future studies should evaluate adherence to a gluten-free diet using objective standards such as serological markers, dietitian assessments, or biochemical indicators to support the scale’s clinical validity. Another limitation is that recruitment through online platforms may have increased participation among adults who use digital platforms more frequently.

## Figures and Tables

**Table 1:** Sociodemographic and general characteristics of patients.

Variables	Mean ± SD	
Age (years)		
Mean ± SD	35.4 ± 11.64	
Age at diagnosis (years)		
Mean ± SD	28.0 ± 12.62	
	**n (97)**	**%**
Gender		
Female	78	80.4
Male	19	19.6
Marital status		
Single	49	50.5
Married	48	49.5
BMI classification (kg/m^2^)		
Underweight	14	14.4
Normal weight	57	58.8
Overweight	16	16.5
Obese	10	10.3
Mean ± SD	22.9 ± 5.01	

SD: Standard deviation, BMI: Body mass index.

**Table 2:** Gluten-free diet expenditure, dietitian visits, and adherence characteristics.

Variables	n (97)	%
Dietitian visits		
Yes	36	37.1
No	61	62.9
Adherence with GFD		
Always	60	61.9
Often	29	29.9
Sometimes	7	7.2
Rarely	1	1.0
Mean ± SD	22.9 ± 5.01	
Adhering to GFD outside		
Always	50	51.5
Often	30	30.9
Sometimes	11	11.3
Rarely	4	4.1
Never	2	2.1
Income spending for GFD		
100%	3	3.1
25%	64	66.0
50%	23	23.7
75%	7	7.2

SD: Standard deviation, GFD: Gluten-free diet.

**Table 3:** Item Statistics of the CDAT.

Items		Mean ± SD
1.	Have you been bothered by low energy level during the past 4 weeks?	3.3 ± 0.96
2.	Have you been bothered by headaches during the past 4 weeks?	2.7 ± 0.97
3.	I am able to follow a gluten free diet when dining outside my home.	2.1 ± 1.8
4.	Before I do something I carefully consider the consequences.	1.6 ± 0.81
5.	I do not consider myself a failure.	2.0 ± 1.21
6.	How important to your health are accidental gluten exposures?	1.7 ± 1.24
7.	Over the past four weeks how many times have you eaten foods containing gluten on purpose?	1.7 ± 1.00

SD: Standard deviation, CDAT: Celiac Dietary Adherence Test.

**Table 4:** Exploratory factor analysis of the CDAT.

Items		Factor Loading
1.	Have you been bothered by low energy level during the past 4 weeks?	0.469
2.	Have you been bothered by headaches during the past 4 weeks?	0.364
3.	I am able to follow a gluten free diet when dining outside my home.	0.717
4.	Before I do something I carefully consider the consequences.	0.465
5.	I do not consider myself a failure.	-
6.	How important to your health are accidental gluten exposures?	0.394
7.	Over the past four weeks how many times have you eaten foods containing gluten on purpose?	0.846

CDAT: Celiac Dietary Adherence Test.

**Table 5:** Confirmatory factor analysis of CDAT.

Fit Indices	Values of CDAT	Acceptable Value
χ^2^/df	1.452	<5
GFI	0.941	0.90 ≤ GFI ≤ 0.95
AGFI	0.881	0.85 ≤ AGFI ≤ 0.90
CFI	0.931	0.90 ≤ CFI ≤ 0.95
NFI	0.819	0.90 ≤ NFI ≤ 0.95
RMSEA	0.068	0.05 ≤ RMSEA ≤ 0.10
TLI	0.896	0.90 ≤ TLI ≤ 0.95

CDAT: Celiac Dietary Adherence Test, GFI: Goodness-of-Fit Index, AGFI: Adjusted Goodness-of-Fit Index, CFI: Comparative Fit Index, NFI: Normed Fit Index, RMSEA: Root Mean Square Error of Estimation, TLI: Tucker–Lewis Index.

**Table 6:** Exploratory factor analysis results of the final 5-item Turkish CDAT.

Items		Factor Loading
1.	Have you been bothered by low energy level during the past 4 weeks?	0.580
2.	Have you been bothered by headaches during the past 4 weeks?	0.560
3.	I am able to follow a gluten free diet when dining outside my home.	0.750
4.	Before I do something I carefully consider the consequences.	0.640
5.	Over the past four weeks how many times have you eaten foods containing gluten on purpose?	0.790

**Table 7:** Internal consistency of the CDAT.

Items	Corrected Item-Total Correlation	Cronbach’s α if Item Deleted	Cronbach’s α
1.	0.304	0.626	0.70
2.	0.308	0.625	
3.	0.550	0.543	
4.	0.404	0.604	
5.	0.187	0.669	
6.	0.231	0.657	
7.	0.616	0.533	

CDAT: Celiac Dietary Adherence Test.

**Table 8:** Test–retest reliability of the CDAT.

Variable	Cronbach’s α	Test ( $\bar{x}$ ± SS)	Retest ( $\bar{x}$ ± SS)	*p* Value (*t*-Test)	ICC (%95 GA)	r	*p* Value (Correlation)
Total score	0.974	12.96 ± 3.45	12.95 ± 3.33	0.925	0.950 (0.926–0.966)	0.950	<0.001

CDAT: Celiac Dietary Adherence Test.

## Data Availability

Data sets used and/or analyzed during the current study are available from the corresponding author. The data is not publicly accessible due to ethical reasons.
